# Einfluss der Distanz zum Thrombus bei akutem Verschluss der Arteria cerebi media: Ein Prädiktor für das klinische Outcome nach mechanischer Thrombektomie

**DOI:** 10.1007/s00117-020-00738-7

**Published:** 2020-08-21

**Authors:** Ruben Mühl-Benninghaus, Salman Nebilir, Andreas Simgen, Gudrun Wagenpfeil, Michael Kettner, Mathias Fousse, Wolfgang Reith, Umut Yilmaz

**Affiliations:** 1 Abteilung für diagnostische und interventionelle Neuroradiologie, Universitätsklinikum des Saarlandes, 66424 Homburg, Deutschland; 2 Institut für Medizinische Biometrie, Epidemiologie und Medizinische Informatik, Universitätsklinikum des Saarlandes, Homburg, Deutschland; 3 Abteilung für Neurologie, Universitätsklinikum des Saarlandes, Homburg, Deutschland

**Keywords:** Gefäßokklusion, Schlaganfall, Mechanische Thrombektomie, CT-Angiographie, Intravenöse Thrombolyse, Vascular occlusion, Stroke, Mechanical thrombectomy, CT angiography, Intravenous thrombolysis

## Abstract

**Hintergrund:**

Die Therapiestrategie von Patienten mit akutem Schlaganfall der Arteria cerebri media (ACM) wird durch die Lokalisation der Okklusion beeinflusst. Diese Studie zielte darauf ab, die klinischen Ergebnisse bei Patienten mit akutem ischämischem ACM-Verschluss, die mit endovaskulärer mechanischer Thrombektomie (EVT) behandelt wurden, entsprechend dem Okklusionsort zu analysieren.

**Methoden:**

Es wurden 54 Patienten (Alter: 73 ± 15 Jahre; 59 % weiblich), die aufgrund eines akuten ACM-Verschlusses mittels EVT behandelt wurden, eingeschlossen. In koronar reformatierten MIP-Bildern (Maximumintensitätsprojektion) der CT-Angiographie wurde die Distanz zum Thrombus (DT), also dem Abstand vom Karotis‑T zum Beginn des Thrombus, gemessen. Die Korrelation zwischen DT, klinischer Symptomatik und klinischem Outcome der Patienten mit EVT-Therapie wurde analysiert.

**Ergebnisse:**

Die DT korrelierte mit der klinischen Symptomatik, gemessen an der National Institutes of Health Stroke Scale (NIHSS; *p* = 0,017; R = −0,324), bei Aufnahme. Sie korrelierte auch mit der modifizierten Rankin-Skala nach 90 Tagen (90-Tage-mRS; *p* = 0,014; R = −0,333). Die DT ist ein Prädiktor für ein gutes klinisches Outcome (mRS nach 90 Tagen) nach EVT; Odds-Ratio: 1,113 (*p* = 0,02; 95 % Konfidenzintervall [KI] 1,017–1,219). Eine DT >10 mm korrelierte signifikant (*p* = 0,036) mit einem guten klinischen Outcome (90-Tage-mRS ≤2).

**Schlussfolgerung:**

Die DT korreliert mit der klinischen Symptomatik von Patienten mit akutem ACM-Verschluss. Darüber hinaus sie ein unabhängiger Prädiktor für das klinische Outcome von Patienten, die an einem akuten Schlaganfall durch ACM-Okklusion leiden.

## Abstract (en)

**Background:**

The therapy strategy of patients with acute stroke of the middle cerebral artery (MCA) is influenced by the location of the occlusion. The purpose of this study was to analyze the clinical outcome in patients with acute ischemic MCA occlusion according to the location of occlusion who underwent endovascular treatment (EVT).

**Methods:**

A total of 54 patients (age 73 ± 15 years; 59% female) with acute ACM occlusion treated with EVT were included. In coronary reformatted CT angiography images, the distance to the thrombus (DT), i.e. the distance from the carotid T to the beginning of the thrombus, was measured. Correlations between DT, clinical symptoms, and clinical outcome of patients who underwent EVT were analyzed.

**Results:**

DT correlated with clinical symptoms measured by the National Institutes of Health Stroke Scale (NIHSS; *p* = 0.017; R = −0.324) at baseline. DT also correlated with the modified Rankin scale after 90 days (90-day mRS; *p* = 0.014; R = −0.333). DT was a predictor for a good clinical outcome (mRS after 90 days) after EVT; odds ratio 1.113 (*P* = 0.02; 95% confidence interval [CI] 1.017–1.219). A DT >10 mm correlated significantly (*p* = 0.036) with a good clinical outcome (90-day mRS ≤2).

**Conclusion:**

DT correlates with the clinical symptoms of patients with acute MCA occlusion. In addition, DT is an independent predictor of the clinical outcome of patients suffering from acute stroke due to MCA occlusion.

Der ischämische Schlaganfall ist eine der Hauptursachen für Tod und körperliche Beeinträchtigung [3]. Die intravenöse Thrombolyse (IVT) mit rekombinantem Gewebeplasminogenaktivator gehört derzeit zur Standardbehandlung für den akuten ischämischen Schlaganfall [9]. Zuletzt konnte gezeigt werden, dass andere akute Therapien, wie die mechanische Thrombektomie, eine vielversprechende Therapieoption darstellen. Eine der bedeutendsten Studien ist die MR-CLEAN-Studie (multizentrische randomisierte klinische Studie zur endovaskulären Behandlung von akutem ischämischem Schlaganfall). Hier konnte gezeigt werden, dass die endovaskuläre mechanische Thrombektomie (EVT) der alleinigen intravenös applizierten Lysetherapie (IVT) bei langstreckigen Gefäßverschlüssen überlegen ist (gutes klinisches Outcome [mRS 0–2] 32,6 vs. 19,1 %; [2]). Für die Entscheidung der Lysetherapie und die Erfolgsaussicht, durch diese Therapie den Thrombus im Gefäß aufzulösen, wurden unterschiedliche Einflussfaktoren untersucht. Wichtige Faktoren für den Erfolg der Thombolyse ist einerseits die Lokalisation des Thrombus und andererseits die Thrombuslänge. Ein proximaler Verschluss oder ein langer Thrombus scheint mit einem ungünstigen Rekanalisationsergebnis zu korrelieren [5, 10, 11]. Sollte bei einem akuten ischämischen Gefäßverschluss die Entscheidung zur EVT als primäre Behandlungsform getroffen werden, so gilt zurzeit die supportative IVT als sog. Bridging-Therapie [8].

Kürzlich wurde von Friedrich et al. der Einfluss der „distance to thrombus“ (Distanz zum Thrombus, DT; d. h. der gemessene Abstand vom Karotis‑T zum Thrombus) von Patienten mit akutem Verschluss der Arteria cerebri media (ACM), die mit IVT behandelt wurden, auf deren klinisches Outcome nach 90 Tagen untersucht.

Ziel dieser Studie war es, den Einfluss der DT von Patienten mit akutem ACM-Verschluss, die mit EVT behandelt wurden, auf das klinische Outcome nach 90 Tagen zu untersuchen.

## Methoden

Diese Studie wurde von unserem lokalen Ethikkomitee genehmigt (Nr. 12/17).

Patienten, die zwischen dem 1. Januar 2014 und dem 30. April 2018 von unserer Einrichtung wegen eines ischämischen Schlaganfalls behandelt worden waren, wurden konsekutiv, retrospektiv auf Ein‑/Ausschlusskriterien hin untersucht.

### Einschlusskriterien

Einschlusskriterien für eine Teilnahme an der Studie waren:

- CT-Angiographie (CTA) – nachgewiesener akuter Gefäßverschluss in der ACM.
- Dokumentation des klinischen Verlaufs:
  - anfänglicher National Institutes of Health Stroke Scale-Score (NIHSS) ≥6
  - anfänglicher modifizierter Rankin Skala-Score (mRS) und nach 90 Tagen (90d-mRS).

### Ausschlusskriterien

Ausgeschlossen wurde Patienten, die eine zusätzliche Vaskulopathie in einem anderen intrakraniellen Gebiet oder in den Halsgefäßen (insbesondere keine relevante vorhergehende Stenose) hatten.

### Bildanalyse

**Figure 1:**
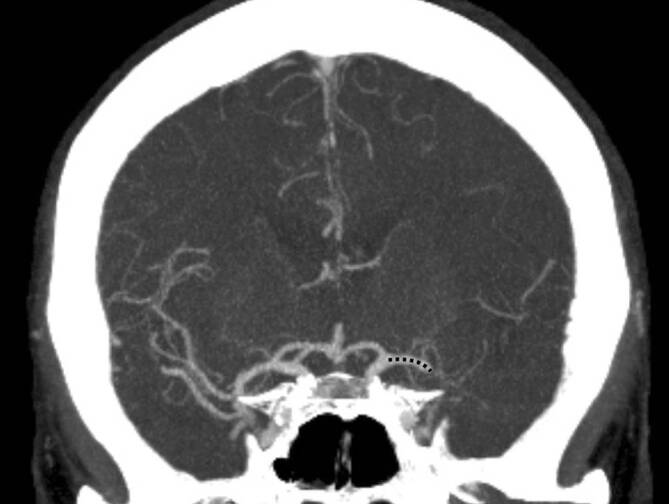

Alle CTA-Untersuchungen wurden unter Verwendung eines Multidetektorscanners mit 32 Abschnitten (Aquilion 32; Toshiba Medical Systems, Tokyo, Japan) mit einer Schichtdicke von 1 mm durchgeführt. CTA-Bilder wurden in der koronaren Ebene rekonstruiert, und ein Projektionsbild mit maximaler Intensität von 10 mm wurde unter Verwendung der Software SectraIDS7 (Sectra, Linköping, Schweden) gerendert. Der genaue Ort der Okklusion wurde bestimmt, und die DT wurde als Abstand vom Zentrum des Karotis‑T zum Beginn des Thrombus gemessen. Das Zentrum des Karotis‑T wurde als Kreuzungspunkt der virtuellen Verlängerung der ACM, der inneren Halsschlagader und der proximalen vorderen Hirnarterie definiert. Die DT wurde als gekrümmte Linie entlang der Mitte der ACM gemessen (Abb. 1). Die DT-Analyse wurde von einem erfahrenen Neuroradiologen (RMB, mindestens 7 Jahre neuroradiologische Erfahrung) durchgeführt. Der Untersucher war während der Durchführung der Messungen für alle Patientenmerkmale vollständig verblindet.

### Statistik

Die statistische Analyse der Daten erfolgte mittels BM SPSS 25 (IBMM, Armonk, New York, USA). Für alle nominalen Daten erfolgte die deskriptive Darstellung anhand ihrer Häufigkeit, ordinalskalierte Daten wurden mittels des arithmetischen Mittelwerts und der Standardabweichung dargestellt. Ob eine Normalverteilung der Daten vorliegt, wurde mithilfe des Shapiro-Wilk-Tests überprüft. Quantitative Merkmale wurden bei vorliegender Normalverteilung mit dem t‑Test verglichen, ansonsten wurde der Mann-Whitney-U-Test verwendet. Für den Vergleich von qualitativen Merkmalen wurde der χ^2^-Test herangezogen. Korrelationen zwischen klinischen Parametern und der DT wurden durch die Verwendung der Korrelationsanalyse mithilfe des Spearman p dargestellt. Die Wahrscheinlichkeit eines guten Outcomes wurde mithilfe der logistischen Regression und der Angabe der Odds-Ratio mit einem Konfidenzintervall von 95 % berechnet. Das Signifikanzniveau wurde mit 5 % festgesetzt, ein *p* ≤ 0,05 galt dementsprechend als statistisch signifikant.

## Ergebnisse

Von 387 wurden 204 Patienten wegen fehlendem 90-Tages-mRS und 129 Patienten wegen zusätzlicher Vaskulopathie in einem anderen intrakraniellen Gebiet oder in den Halsgefäßen ausgeschlossen. Insgesamt 54 Patienten (Alter: 73 ± 15 Jahre; 59 % weiblich) wurden in die Studie eingeschlossen. Weitere demographische Merkmale der Patienten sind in Tab. 1 aufgeführt.

**Table 1:** 

Anzahl der Patienten	54
Weiblich, *n* (%)	33 (59)
Alter, Mittelwert ± SD	73 ± 15
NIHSS bei Aufnahme ± SD	15 ± 8
mRS vor Schlaganfall, median (IQR)	0 (0–0)
mRS bei Aufnahme, Mittelwert ± SD	4,4 ± 0,9
mRS 90 Tage nach Intervention, Mittelwert ± SD	2,7 ± 2
Bridging (IVT + EVT), *n* (%)	36 (67)
*Risikofaktoren, n (%)*	
Koronare Herzkrankheit	12 (22)
>1 Schlaganfall in der Vorgeschichte	11 (20)
Arterielle Hypertension	44 (82)
Diabetes mellitus	13 (24)
Nikotinabusus	Nicht eruierbar
Alkoholabusus	Nicht eruierbar
Hyperlipoproteinämie	12 (22)
*TOAST-Klassifikation, n (%)*	
Unklare Ursache	14 (26)
Kardiale Embolie	34 (63)
Arteriosklerose	5 (9)
Andere Ursache	1 (2)
Zeit zwischen Aufnahme und Rekanalisation, min., ±SD	105 ± 51
Dauer der EVT, min., ±SD (Zeit: Leistenpunktion bis Rekanalisation)	27,17 ± 21,14

*EVT* endovaskuläre Thrombektomie, *IVT* intravenöse Thrombolyse, *NIHSS* National Institutes of Health Stroke Scale, *SD* Standardabweichung, *mRS* modified Rankin-Skala, *TOAST* Trial of Org 10172 in Acute Stroke Treatment

**Figure 2:**
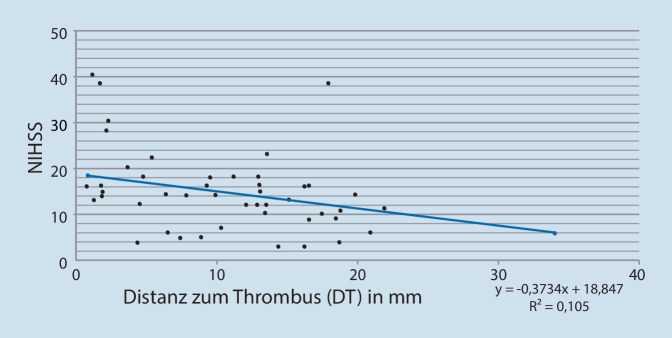

**Figure 3:**
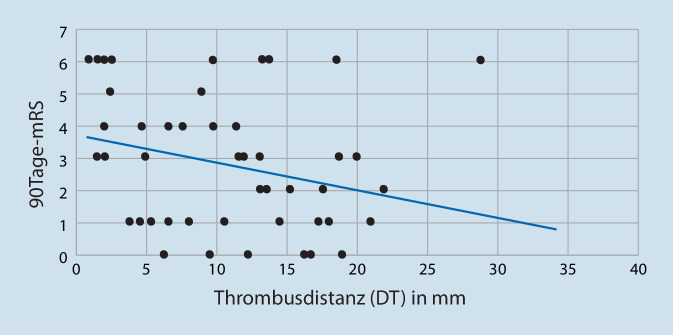

Eine genaue Lokalisation der Okklusion sowie eine Bestimmung des DT war bei allen Patienten im CT möglich. Die DT wurde wie in Abb. 1 dargestellt gemessen, und die mittlere DT lag bei 11,3 ± 7,2 mm. Die demographischen Daten der Patienten sind in Tab. 1 beschrieben. Die Bivariat-Korrelationsanalyse nach Spearman zeigt eine statistisch signifikante Korrelation des DT mit dem NIHSS (*p* = 0,017; R = −0,324; Abb. 2). Auch zwischen der mRS nach 90 Tagen und DT (Abb. 3) wurde eine signifikante Korrelation (*p* = 0,014, R = −0,333) beobachtet.

**Figure 4:**
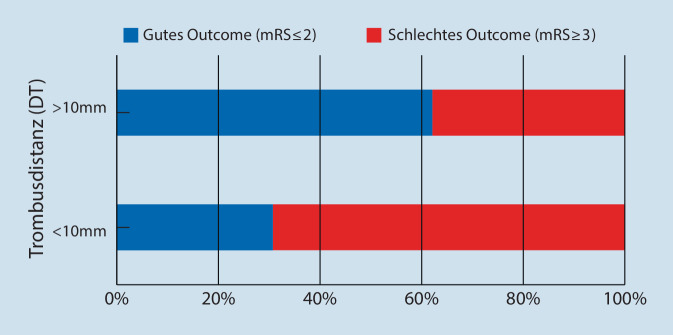

Die binär logistische Regression zeigte, dass die DT ein Prädiktor für ein gutes klinisches Outcome (mRS ≤2) nach 90 Tagen war (Odds-Ratio von 1,113; *p* = 0,02, 95 % KI 1,017–1,219). Patienten mit einer mittleren DT ≤10 mm hatten einen signifikant niedrigeren 90-Tage-mRS (Abb. 4) als Patienten mit einer DT >10 mm (*p* = 0,036).

## Diskussion

Unsere Untersuchungen zeigten, dass die DT ein hochsignifikanter Prädiktor für das klinische Ergebnis bei Patienten mit akutem ACM-Verschluss war, die mit EVT behandelt wurden.

Der Nutzen der Behandlung eines Schlaganfalls mit IVT und EVT konnte in verschiedenen multizentrischen Studien gezeigt werden [4, 6, 7, 12]. Die meisten dieser Studien haben die genaue Lokalisation der Okklusion innerhalb der ACM jedoch nicht in ihren Analysen oder Einschlusskriterien berücksichtigt.

Andere Studien hingegen konnten allerdings den Einfluss der genauen Lokalisation der ACM-Okklusion in der Angiographie (CTA oder digitale Subtraktionsangiographie) auf das klinische Outcome von Patienten mit ischämischem Schlaganfall nachweisen. So wurde berichtet, dass die Wahrscheinlichkeit für ein ungünstiges klinisches Outcome von Patienten, die mit IVT oder EVT behandelt wurden, mit einer proximaleren Okklusion, d. h. einer geringeren DT zunimmt [1, 5, 11]. Rohan et al. [2] verwendeten eine diffizile Analysemethode zur Berechnung der DT, die auf der Auswertung von 4‑D-CTA-Bildern basiert und im klinischen Alltag schwer umzusetzen ist. Behme et al. [1] teilten lediglich den Ort der M1-Okklusion in 2 Gruppen auf, sodass die genaue DT hier nur indirekt berücksichtigt wurde.

Die Erkenntnisse der o. g. Studien belegen die Wichtigkeit der genauen Lokalisation des Gefäßverschlusses innerhalb der ACM bei Patienten mit akutem ischämischem Schlaganfall, um Prognosen abzuschätzen oder therapeutische Entscheidungen zu treffen. Unsere Messmethode basiert auf der Methode von Friedrich et al. [5], die sie bei Patienten mit IVT-Behandlung bei akuter ACM-Okklusion anwendeten, da sie einfach anwendbar und genauer als die o. g. Messmethoden ist. In unserer Analyse zeigte sich, dass Patienten mit einer DT >10 mm ein signifikant besseres Outcome nach 90 Tagen hatten. Im Gegensatz dazu beschrieben Friedrich et al. [5], dass ein gutes klinisches Outcome erst ab einer DT >16 mm zu erwarten ist. Diese unterschiedlichen Ergebnisse können daran liegen, dass wir die EVT anstelle der IVT als Behandlungsform angewandt haben. Zusätzlich kann auch die *Bridging-Therapie* (IVT + EVT), welche die meisten Patienten (67 %) in unserer Studie erhielten, Einfluss auf die unterschiedlich gemessene DT in den Studien gehabt haben.

Unsere Ergebnisse in Kombination mit anderen veröffentlichten Daten legen jedoch nahe, dass das klinische Outcome sowohl nach IVT als auch nach EVT in Abhängigkeit der Lokalisation der Okklusion variiert.

Die Aussagekraft dieser Studie ist durch das retrospektive Design eines einzelnen Zentrums und die relativ geringe Anzahl von Patienten limitiert. Eine weitere Einschränkung besteht darin, dass nur ein erfahrener Neuroradiologe die DT-Werte gemessen hat und daher die Zuverlässigkeit zwischen den Untersuchern nicht beurteilt werden konnte.

## Fazit für die Praxis

- Die Distanz zum Thrombus korreliert mit der klinischen Symptomatik von Patienten mit akutem ACM-Verschluss.
- Darüber hinaus ist sie ein unabhängiger Prädiktor für das klinische Outcome von Patienten, die an einem akuten Schlaganfall durch ACM-Okklusion leiden.

## Abkürzungen

ACM: Arteria cerebri media
CTA: CT-Angiographie
DT: Distanz zum Thrombus
EVT: Endovaskuläre mechanische Trombektomie
IVT: Intravenöse Thrombolyse
MR-CLEAN: Multicenter randomized clinical trial of endovascular treatment for acute ischemic stroke in the Netherlands
mRS: Modifizierte Rankin-Skala
NIHSS: Institutes of Health Stroke Scale
TOAST: Trial of Org 10172 in Acute Stroke Treatment
